# Characterization of Breast Cancer Aggressiveness by Cell Mechanics

**DOI:** 10.3390/ijms241512208

**Published:** 2023-07-30

**Authors:** Barbara Zbiral, Andreas Weber, Maria dM. Vivanco, José L. Toca-Herrera

**Affiliations:** 1 Institute of Biophysics, Department of Bionanosciences, University of Natural Resources and Life Sciences, Muthgasse 11, 1190 Vienna, Austria; barbara.zbiral@students.boku.ac.at (B.Z.); andreas.weber@bokuac.at (A.W.); 2 Cancer Heterogeneity Lab, Center for Cooperative Research in Biosciences (CIC bioGUNE), Basque Research and Technology Alliance (BRTA), Bizkaia Technology Park, 48160 Derio, Spain; mdmvivanco@cicbiogune.es

**Keywords:** breast cancer, cancer cell mechanics, atomic force microscopy, viscoelasticity, cell adhesion, cytoskeleton

## Abstract

In healthy tissues, cells are in mechanical homeostasis. During cancer progression, this equilibrium is disrupted. Cancer cells alter their mechanical phenotype to a softer and more fluid-like one than that of healthy cells. This is connected to cytoskeletal remodeling, changed adhesion properties, faster cell proliferation and increased cell motility. In this work, we investigated the mechanical properties of breast cancer cells representative of different breast cancer subtypes, using MCF-7, tamoxifen-resistant MCF-7, MCF10A and MDA-MB-231 cells. We derived viscoelastic properties from atomic force microscopy force spectroscopy measurements and showed that the mechanical properties of the cells are associated with cancer cell malignancy. MCF10A are the stiffest and least fluid-like cells, while tamoxifen-resistant MCF-7 cells are the softest ones. MCF-7 and MDA-MB-231 show an intermediate mechanical phenotype. Confocal fluorescence microscopy on cytoskeletal elements shows differences in actin network organization, as well as changes in focal adhesion localization. These findings provide further evidence of distinct changes in the mechanical properties of cancer cells compared to healthy cells and add to the present understanding of the complex alterations involved in tumorigenesis.

## 1. Introduction

Breast cancer is the most diagnosed cancer worldwide. In the year 2020, an estimated 2.3 million new cases of breast cancer were diagnosed globally, with 685,000 breast cancer deaths recorded. With 16% of cancer deaths corresponding to breast cancer, it accounts for the largest fraction of cancer-related deaths in women and is predicted to continue increasing to over 3 million new cases by 2040 [1]. Breast cancer is a heterogenous disease that can be classified into various subtypes, which are associated with different clinical outcomes [2]. Approximately 70% of diagnosed cancers express estrogen and progesterone receptors (ER+/PR+). Thus, hormone therapy with tamoxifen, and more recently with aromatase inhibitors, is commonly the intervention of choice [3,4]. Unfortunately, the development of resistance to hormone therapy, even years after stopping the treatment [5], poses a clinical challenge by narrowing down the available treatment options and worsening disease prognosis [6,7]. Notably, HER2 overexpression (HER2+) in breast cancer is correlated with faster tumour growth and higher risk of recurrence, however, it also provides a specific target for antibody-based cancer therapy with, e.g., trastuzumab (Herceptin) [8,9,10]. A breast cancer subtype that no longer express functional hormone receptors (ER-/PR-) or HER2 is called triple-negative breast cancer (TNBC). Epidemiologically, TNBC is overrepresented in black women and pre-menopausal (<40-year-old) women and makes up an estimated 10–20% of breast cancers, with increasing incidence [11,12]. TNBC poses the clinical challenge of lacking a tumour-specific drug target, curtailing avenues for drug therapy, aside from cytotoxic chemotherapy or novel antibody-drug-conjugates, such as Sacituzumab govitecan [13]. As a result, these cancers are regarded as particularly aggressive, since they are known to be more invasive and have a higher inclination to metastasize, as well as a higher recurrence rate after treatment, compared to other breast cancer subtypes [11,12,14,15,16].

Generally, the threshold separating a benign tumour from a malignant lesion is the ability to invade distant tissues and metastasize [17]. Once a carcinoma becomes invasive, treatment success rates diminish [18,19]. Both TNBC and tamoxifen-resistant ER+ subtypes upregulate cellular markers associated with invasiveness as signals that modulate cell adhesive properties, cell-to-cell contacts, and cytoskeletal arrangement [20]. The latter in particular plays an integral role in modulating cell mechanical properties. Cells feel and respond to forces of their environments, such as traction, shear, and compression. In turn, they pull and push onto their surrounding microenvironment and neighbouring cells. Healthy tissue exists in a mechanical homeostasis, as cellular forces are important signalling factors decisive in cell fate during differentiation, growth, and tissue modulation. While the dynamic remodeling of the ECM is part of normal tissue maintenance, excessive or uncontrolled tissue remodeling contributes to and results from diseases like cancer, where cell division happens uncontrolled and at an exaggerated rate. Cell crowding causes increased local pressure in the affected tissue, forcing cells within the lesion to adapt their own mechanical properties to withstand their environment [21,22,23].

Oncogenes engage signalling pathways to enhance intracellular tension via increased actomyosin contractility and adapt the mechanical phenotype of the cell by altering gene expression [21]. Research shows that breast epithelial cells cultured in ECMs of physiological stiffness retain their normal phenotype but display structural and transcriptional hallmarks associated with tumorigenicity when cultured in ECMs of a stiffness resembling tumour stroma [24]. The processing of these mechanical signals from the microenvironment involves integrin clustering, ERK activation, cytoskeletal remodeling, and Rho GTPase-dependent contractility. Thus, growth factor signalling, mechanotransduction, cytoskeletal arrangement, as well as adhesion and contraction are functionally tightly linked [24,25]. With the cumulative understanding of the significance of mechanobiological changes during malignant progression growing, some researchers suggest that a distinct “mechanotype” for cancer cells exists [26]. As cells progress from normal, to preinvasive, to invasive cells, cell stiffness decreases, and deformability increases gradually. Thus, the mechanotype pertains to cancer cells undergoing malignant transformation and is considered a hopeful tool for a label-free biomarker of cell state that could represent an additional approach to cancer cell classification [26,27].

Nowadays, many techniques specialized in characterizing biological materials by their mechanical parameters are established. Different methods probe cell mechanics on a different hierarchical or organizational scale and provide distinct sets of advantages and drawbacks. As such, elastic and viscoelastic properties of cells and tissues have been derived from experiments employing methods like magnetic or optical tweezers, bead-based microrheology, micropipette aspiration and atomic force microscopy [28,29,30,31,32,33,34,35]. Here, we use atomic force microscopy (AFM)-based force spectroscopy to determine the cell mechanics of various breast cancer cell lines that represent different breast cancer subtypes with various degrees of aggressiveness. We characterise four different cell lines for their elastic and viscoelastic properties. Non-tumorigenic MCF10A cells, a non-invasive breast epithelial cell line that serves as a model for normal human breast cells. MDA-MB-231 cells represent a TNBC subtype with a high degree of tumorigenicity and invasiveness. MCF-7 breast cancer cells are malignant and a representative example of the ER+/PR+ breast cancer subtypes, which have better prognosis than hormone-independent tumours [36]. MCF-7TamR cells are a subvariant of MCF-7 cells that have developed resistance to the anti-estrogen tamoxifen, a commonly used hormone therapy to treat ER+ breast cancer, and show increased invasion capacity, CSC content and tumorigenicity [36,37]. As MCF-7TamR cells are derived from hormone-sensitive MCF-7 cells, they should primarily be compared to MCF-7 cells when considering cell mechanical and structural properties. The non-tumorigenic MCF10A cells serve as healthy controls for MCF-7 and MDA-MB-231 breast cancer cells. We have determined Young’s moduli via indentation studies from a modified Hertz mechanical model and viscoelastic moduli as well as the viscosities of the cell lines through stress–relaxation experiments via a five-element general Maxwell (Zener’s) model [37]. Confocal laser scanning microscopy images with fluorescently stained actin, tubulin, vinculin, and nuclei were acquired to visualize the differences in subcellular organization for the differently (aggressive) cell lines. AFM experiments show which cell lines tend to be softer, while CLSM images correlates such results with structural differences in actin arrangement.

## 2. Results

### 2.1. Increased Malignancy Correlates with Cell Softening

#### 2.1.1. Elastic Parameters

Cells were exposed to an applied force of 1 nN. The apparent Young’s moduli and the cell deformations at that force were determined from the recorded force curves.

**Figure 1:**
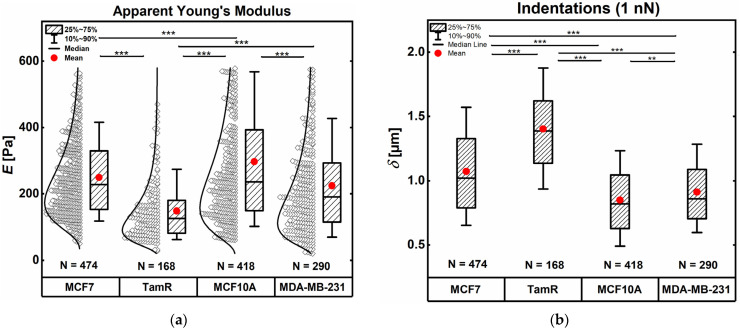
Cell mechanical data derived from the indentation segments of AFM force spectroscopy curves: (**a**) elasticity *E* expressed as the apparent Young’s modulus and (**b**) indentations recorded at 1 nN for the examined cell lines. Boxes range from 25th to 50th, and whiskers from 10th to 90th percentiles. For raw data display in (**a**), a bin size of 10 Pa was chosen. Outliers were omitted, ‘**’ indicates a *p*-value of <0.01 and ‘***’ indicates a *p*-value < 0.001.

Figure 1a represents the derived apparent elastic moduli of the different cell lines. Non-malignant and non-invasive MCF10A cells showed the highest mean apparent Young’s modulus at 279 Pa and can thus be considered the stiffest of the studied cell lines. The next stiffest cell line was MCF-7, which are cancer cells with low invasive ability, with a mean apparent Young’s modulus of 249 Pa (−11% compared to MCF10A cells). Highly invasive MDA-MB-231 cancer cells were even softer, averaging a Young’s modulus of 224 Pa (−20% compared to MCF10A cells). Tamoxifen resistant-MCF-7 (TamR) cells appeared to be the softest cell line with a mean Young’s modulus of 148 Pa, making them approximately 40% softer than their respective parental MCF-7 cancer cells, and almost 50% softer than the healthy MCF10A cells.

Figure 1b shows the corresponding cell deformation at the applied force of 1 nN. In accordance with the highest mean apparent Young’s modulus, MCF10A cells were deformed the least at the applied force, following the expected trend of inverse correlation between deformation and cell stiffness. For MCF-7 and MCF-7TamR cells, this trend was consistent as well. Interestingly, MDA-MB-231 cell deformation under the given load was slightly smaller than the deformation of the MCF10A cells, despite their elastic stiffness being on average 20% lower than that of MCF10A. MCF-7TamR cell indentation increased by roughly 27% compared to non-resistant MCF-7 cells.

#### 2.1.2. Viscoelastic Parameters

Living cells behave as viscoelastic materials due to their heterogenous internal structure. Thus, elastic parameters alone do not sufficiently describe the material properties of a living cell and studying the time-dependent force response of cells is required. Thus, the viscoelastic properties of the cells were examined by loading cells to 1 nN and recording their stress relaxation response over 10 s.

**Figure 2:**
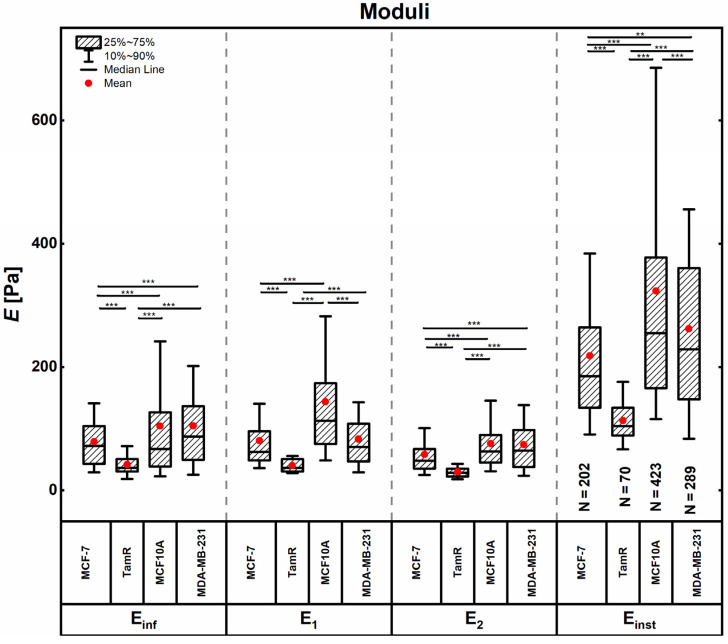
Values of the moduli derived from stress–relaxation experiments. Boxes range from 25th to 50th, and whiskers from 10th to 90th percentiles. Outliers were omitted, ‘**’ indicates a p-value < 0.01 and ‘***’ indicates a *p*-value < 0.001.

**Figure 3:**
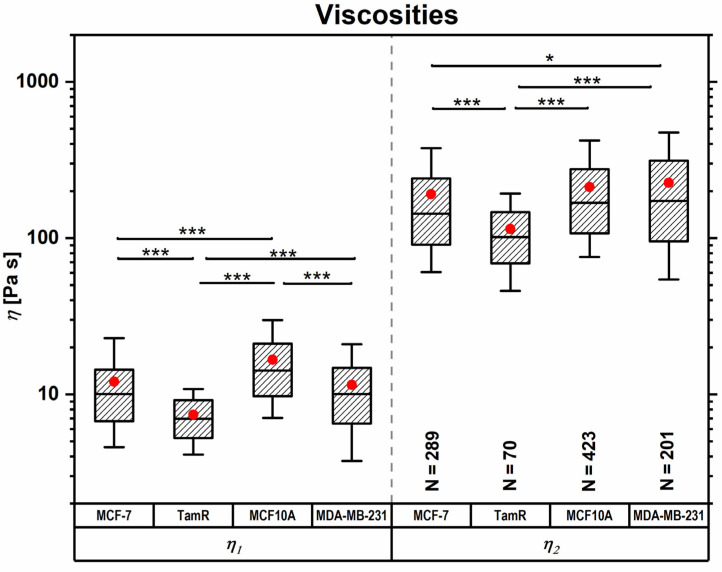
Cell viscosities derived from two distinct stress–relaxation timescales, corresponding to different subcellular compartments. Boxes range from 25th to 50th, and whiskers from 10th to 90th percentiles. Outliers were omitted, ‘*’ indicates a *p*-value < 0.5 and ‘***’ indicates a *p*-value < 0.001.

A five-element Maxwell model (Zener’s model; see Materials and Methods section) was used to determine the (elastic) moduli shown in Figure 2 and the viscosities shown in Figure 3, by fitting a double-exponential decay function to the stress relaxation segment of the force spectroscopy curves. The fit yields three elastic moduli and two distinct relaxation times, a short-term relaxation time of <1 s and a long-term relaxation time of <10 s. Taking the cell deformations (indentations δ) into consideration, moduli and viscosities can be calculated according to the mechanical model. The equilibrium modulus *E*_inf_ corresponds to the single spring element of the Maxwell model and represents the remaining elasticity after the complete stress relaxation of the cell, while moduli *E*_1_ and *E*_2_ describe the springs in series with the viscous dampener elements. The instantaneous modulus *E*_inst_ is the sum of all springs in the system and corresponds to the elastic modulus in this model. The viscosities of the cells *η*_1_ and *η*_2_ are derived from the short-term and long-term relaxation times, *τ*_1_ and *τ*_2_, respectively.

Different subcellular structures are thought to give rise to the different elements of the Maxwell model when responding to external forces, due to their biomechanical properties [38,39,40,41]. The passive structural compartments of the cell, such as the membrane and the cytosol, behave more fluid-like and deform readily and quickly under an applied load. Thus, the shorter relaxation time *τ*_1_ and the derived viscosity *η*_1_, as well as the respective spring element *E*_1_, are considered descriptive of those structures. The longer relaxation time *τ*_2_, partnered with *E*_2_ and the viscosity *η*_2_ are linked with the more solid-like subcellular structure, mainly the cytoskeleton and the actin cell cortex, which rearrange actively and over a longer time frame.

For the membrane- and cytosol-associated modulus *E*_1_ and the instantaneous modulus *E*_inst_, MCF10A cells showed the highest values, followed by MDA-MB-231, MCF-7 and finally MCF-7TamR cells. For the cytoskeleton-associated modulus *E*_2_ and the equilibrium modulus *E*_inf_, MDA-MB-231 and MCF10A cells displayed similar values, followed by MCF-7 and MCF-7TamR cells. Similarly, the viscosities shown in Figure 3 and Table 1 derived from the two distinct relaxation times are thought to indicate the viscosities of the different subcellular structures. For either case, MCF10A cells displayed the highest viscosity, while MCF-7TamR cells appeared the most fluid-like. For the viscosity derived from the shorter time regime, MCF-7 and MDA-MB-231 cells showed similar values. For the viscosity stemming from the longer time regime, thought to arise from cytoskeletal contributions, MDA-MB-231 cells displayed a higher value and MCF-7 cells appeared more fluid like.

All mean calculated values for the mechanical parameters are listed in Table 1 and can be found in detail in Supplementary Table S1.

### 2.2. CLSM Imaging Demonstrates Structural Differences in Cytoskeletal Arrangement

**Figure 4:**
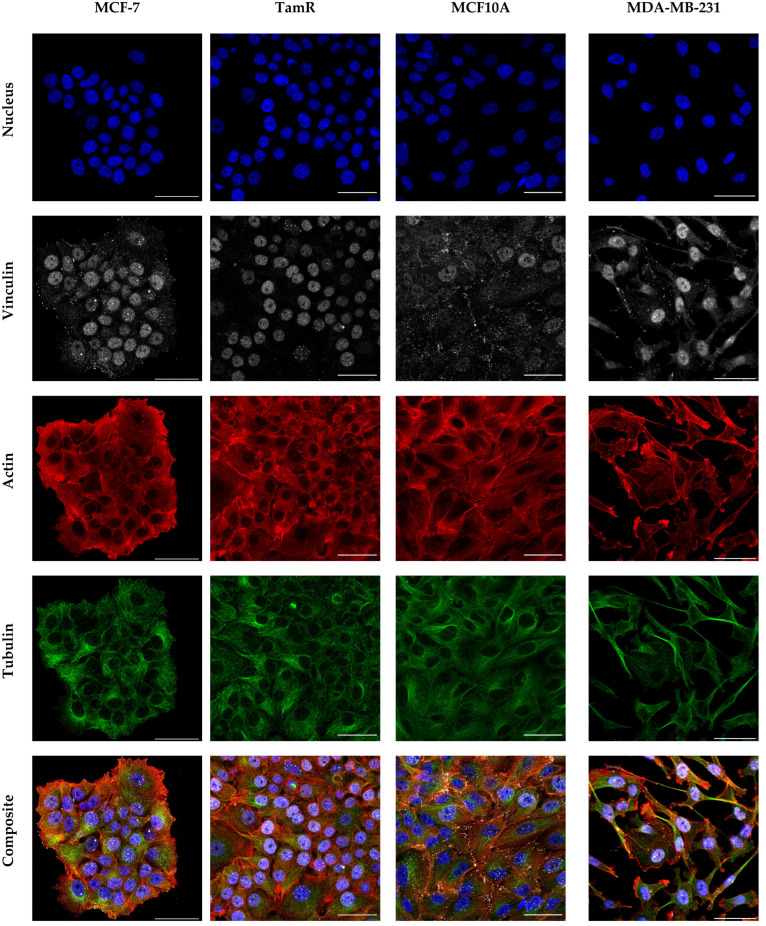
Representative immunofluorescence CLSM images of each of the studies cell lines showing nuclei in blue, vinculin in grey, the actin cytoskeleton in red, microtubules in green and a composite overlay. Notable differences in actin arrangement and vinculin expression between the cell lines are observable. All scale bars correspond to 50 µm.

To visualize the structural differences between these breast cancer cell lines, fluorescence confocal microscopy images were acquired. Nuclei, microtubules, actin, and the focal adhesion protein vinculin were stained with fluorescent dyes. Representative images are found in Figure 4 and in larger size indicated with arrows to highlight respective structural differences in Figures S1–S4 of the Supplementary Information (SI).

MCF-10A cells displayed well-structured cytoskeletal components with most of the actin aligned in parallel stress fibers. Vinculin is found mainly localized in focal adhesions at the ends of these and, to some extent, in the nucleus. In contrast, both MCF-7 and MCF-7TamR cells showed more amorphous actin structures throughout the cell and slightly stronger localization on cell rims and cell-to-cell borders. While MCF-7 cells showed some, albeit few, pronounced actin stress fibers, few to none were observable for MCF-7TamR cells. Instead, actin fibrils on cell borders appearing more diffuse. MDA-MB-231 cells appeared unable to form a tight epithelial sheet. While they displayed well-defined stress fibers across the cell body, albeit far fewer than non-malignant MCF10A, actin was predominantly localized in protrusion-like structures on cell edges, akin to filopodia and lamellipodia [42], which was not associated with focal adhesions. Visually, MDA-MB-231 cells appeared morphologically the most heterogenous of the studied cell lines. All cell lines expressed vinculin in focal adhesions, as well as in the cytosol and the nucleus, to varying degrees. Notably, MCF-7TamR cells showed only very sporadic focal adhesion formation. No remarkable morphological differences in tubulin expression or localization were observable from these analyses.

## 3. Discussion

The mechanical and structural properties of four breast cancer cell lines with different degrees of aggressiveness were studied.

The links between cell mechanics and subcellular structures, such as the cytoskeleton and cancer cell malignancy, are robustly established in mechanobiology research for various tissue and cancer types, with a variety of orthogonal methods used to corroborate these findings [21,25,43,44,45,46,47,48,49,50,51]. The links between invasiveness and cell mechanical alterations have been examined via AFM in the past [48,49]. For example, one study examined the mechanical properties of metastatic cancer cells versus benign mesothelial cells taken from human bodily fluids via AFM and found metastatic cancer cells to be more than 80% softer than benign cells [50]. Cells sense and respond to the mechanical cues of their environment and adapt their cell mechanical properties accordingly. This is integral to tissue homeostasis. We observe progressive cell softening and alterations in viscoelastic properties the more malignant the breast cancer cell line. Cell softening has been associated with invasive capacities of cancer cells, as more pliable cells migrate through tissue more easily [43,51,52,53,54]. The main mechanism found to be responsible is active cytoskeletal remodeling. Our AFM and confocal data agree with the published literature regarding the link between actin rearrangement, degree of malignancy and cell softening.

Out of the examined cell lines, the non-tumorigenic MCF10A cells are the stiffest cell line with well-defined ventral actin stress fibers. MCF-7 cancer cells, which are malignant with low invasive potential, are softer with seemingly fewer and thinner ventral stress fibers, but with more transverse actin arcs forming lamellae [55,56]. The highly invasive, triple-negative MDA-MB-231 cells are softer than both MCF-7 and MCF10A cells, despite forming well-defined ventral actin stress fibers, with few to no transverse arcs. Additionally, these cells form some migratory actin protrusions. When considering their viscoelastic properties, the modulus and viscosity derived from the longer relaxation time are similar to those of MCF10A cells. This might be attributed to the expression of ventral stress fibers in both cell lines. Their comparatively low elastic modulus may thus be due to factors besides actin arrangement. When probing a cell with a spherical particle above the nuclear region, the average mechanical profile of the entire cell is recorded due to the relatively large contact area. Thus, contributions arise not only from cytoskeletal components, but also from the cytoplasm, the cell membrane, and the nucleus. The latter has been implicated in cancer cell migration more recently. Recent research has found the nucleus itself to function as a mechanosensor [57,58]. Moreover, there is evidence that its shape is actively controlled by an actin structure called the perinuclear actin cap [59]. This actin cap actively exerts force on the nucleus to maintain and—in the case of cell migration—modulate its shape to facilitate migration through matrices and tissues [60]. Considering the high migratory potential of MDA-MB-231 cells and the visual heterogeneity of the population, which can be observed in Figure 4, it is possible that the different measured parameters are dominated strongly by different contributions of each cell constituent (for example, MDA-MB-231 cells are more elongated). MCF-7TamR cells are the softest of the examined cell lines. They show no notable ventral stress fiber formation and actin at the cell borders as well as inside the cells appears more diffuse and less well-structured than in MCF-7 cells or any of the other cell lines, which may well explain their significant softening when compared to MCF-7 cells.

In published research, a subset of 629 proteins have been found significantly altered upon acquisition of tamoxifen resistance in MCF-7 cells, including vital players in regulating the cytoskeleton, migratory capacity, survival signalling and estrogen receptor suppression [61]. Notably, while estrogen signalling is downregulated during drug resistance acquisition, ER is often still expressed [36]. As estrogen-dependence decreases, cells have been found to upregulate other pathways to enhance growth signalling. Among those, MAPK and PI3K/Akt increase growth signalling and the latter has been linked to actin cytoskeleton rearrangements and the modulation of cell motility in invasive breast cancer [61,62,63]. Furthermore, enhanced Rho-ROCK signalling has been connected to increased motility in MCF-7TamR cells as well [61]. While canonical ER-dependent signalling is downregulated in MCF-7TamR cells, other ER-independent estrogenic signalling pathways, like GPER (G-protein coupled estrogenic receptor) signalling, might be active. Importantly, GPER activation decreases actin stress fiber thickness, focal adhesion size and number and cytoskeletal stiffness [64].

Focal adhesions influence cell mechanics by modulating processes like cell adhesion, cell spreading and migration [65,66]. Our confocal data regarding vinculin and actin are in line with these findings, as both MCF-7TamR and MDA-MB-231 cells tend to have fewer focal adhesions and thinner actin stress fibers than MCF-7 and MCF10A cells, respectively. Particularly, MCF-7TamR cells form focal adhesions only very sporadically. This is likely linked with their stress fiber expression profile and may suggest a suppressed cell adhesion machinery leading to increased migration and invasion capacity, as previously reported [36]. Indeed, continuous tamoxifen exposure has been shown to induce cytoskeletal remodeling towards the formation of lamellipodia, filopodia and membrane spikes [67]. This also potentially explains the diffused appearance of the actin fibers MCF-7TamR cells show under confocal microscopy. GPER signalling is found in both ER- and ER+ cells and it seems to be involved in a positive feedback loop during the acquisition of tamoxifen resistance [68,69,70]. Tumours that have developed resistance to tamoxifen express higher levels of Sox2 [36] and we have previously reported that Sox2, which is significantly increased in MCF-7TamR cells, alters the cytoskeleton structure from an organized to an irregular network, leading to reduced cell stiffness [71]. Additional studies to further explore the role of cell mechanics in tumour progression and the development of resistance to therapies are warranted. These findings support the view that physical alterations participate in tumour progression and increased aggressiveness.

## 4. Materials and Methods

### 4.1. Cell Culture

MCF-7 and MDA-MB-231 breast cancer cells were obtained from the American Type Culture Collection (ATCC, Manassas, VA, USA) and grown in high glucose DMEM (Gibco, 4.5 g/L glucose, Thermo-Fisher, Vienna, Austria) with L-glutamine, 8% (*v*/*v*) fetal bovine serum (FBS, Gibco, Thermo-Fisher, Vienna, Austria) and 1% (*v*/*v*) Penicillin-Streptomycin (PenStrep, Gibco, Thermo-Fisher, Vienna, Austria). MCF-7TamR cells were derived as previously reported [36] and cultivated in the same medium in the presence of 5 × 10^−7^ M tamoxifen. MCF10A cells were obtained from ATCC (ATCC, Manassas, VA, USA) and cultivated in DMEM/F12 1:1 (Gibco, Thermo-Fisher, Vienna, Austria) medium supplemented with 5% (*v*/*v*) horse serum (Gibco, Thermo-Fisher, Vienna, Austria), 0.5 µg/mL hydrocortisone, 20 ng/mL human epithelial growth factor, 100 ng/mL cholera toxin and 10 µg/mL insulin. In routine culture, cells were passaged twice a week in sub-culturing ratios of 1:6 and cultivated in a 37 °C, 5% CO_2_ and 95% humidity atmosphere.

### 4.2. Atomic Force Microscopy (AFM) Force Spectroscopy

For AFM measurements, cells were grown on Ø 24 mm borosilicate glass coverslips. Before seeding, coverslips were cleaned in 70% ethanol, dried with N_2_ and oxygen-plasma cleaned for 60 s. Coverslips were immediately transferred into the wells of a sterile F-bottom cell culture 6-well plate covered with 1× PBS to minimize loss of surface functionalization. Cells were harvested using TrypLE™ Express (Gibco, Thermo-Fisher, Vienna, Austria) and seeded in their respective culture medium at a density of 1 × 10^5^ cells per well. Samples were incubated overnight at cultivation conditions prior to measurements.

Force spectroscopy measurements were conducted with a JPK Nanowizard III atomic force microscope combined with a JPK CellHesion^®^ module (Bruker, Berlin, Germany), mounted on top of an inverted optical microscope (Axio Observer Z1, Zeiss, Oberkochen, Germany). To emulate cultivation conditions as closely as possible, cell samples were kept in a temperature-controlled liquid-sample chamber at 37 °C in Leibovitz L15 (Gibco, Thermo-Fisher, Vienna, Austria) CO_2_-independent medium. Silica particles with a diameter of 10 µm were glued to tipless, triangular cantilevers (DNP-O, Bruker, Camarillo, CA, USA; nominal spring constant of 0.12 N m^−1^ and resonance frequency of 23 kHz in air) as described previously [37]. Cantilevers were cleaned with EtOH, dried with N_2_ and UV/O-cleaned for 30 min prior to experiments. Before the start of each experiment, the experimental set-up was left to equilibrate for 30 min. At the start of each experiment, the cantilever sensitivity and spring constants were determined as previously described [72]. Cells were approached with a loading rate of 5 µm/s and indented to a set force of 1 nN, then the cantilever height was kept constant for 10 s and the force decay over time (stress relaxation) was recorded. Finally, the cantilever was retracted at a rate of 5 µm/s. The z-range for approach and retract was 50 µm and sampling rates of 1024 Hz for approach and retract, and 512 Hz for the stress relaxation segment, were chosen. Each cell was indented 5x above the nuclear region of the cells, as controlled via the optical microscope. For each sample, at least 30 cells and at least 2 samples per cell line were measured. Samples were kept no longer than 4 h in the liquid sampling chamber; over this timespan no morphological changes were observed.

### 4.3. Confocal Laser Scanning Microscopy (CLSM)

Cells were grown in culture medium in 8-well Ibidi µ-slides at a density of 5 × 10^4^ cells/mL overnight, then fixed with 4% paraformaldehyde (Merck, Darmstadt, Germany) for 10 min at RT and permeabilized with 0.1% Triton X-100 (Merck, Darmstadt, Germany) in PBS at RT for 15 min. For blocking of unspecific epitopes, cells were incubated with 2% BSA in PBS for at least 2 h at RT or in 4 °C overnight. For immunostaining, cells were incubated with primary anti α-tubulin mouse monoclonal antibody (13-1700, Thermo-Fisher, Waltham, MA, USA), diluted 1:1000 in 1% BSA (2 µg/mL final concentration) and rabbit anti-Vinculin primary antibody 1:2000 (Thermo-Fisher, Waltham, MA, USA) at 4 °C overnight. Secondary anti-mouse AlexaFluor 488 and anti-rabbit AlexaFluor 633 conjugated antibodies were added (1:1000 in 1% BSA (2 µg/mL), Thermo-Fisher, Waltham, MA, USA) together with AlexaFluor 555-labeled phalloidin (1:100 in 1% BSA, Thermo-Fisher, Waltham, MA, USA) for 60 min at RT. Nuclei were stained with Hoechst 33,342 (diluted 1:2000 in PBS, Thermo-Fisher, Waltham, MA, USA). Between all steps, cells were washed 3x with 1x PBS. Samples were sealed with IBIDI mounting medium, stored at 4 °C and protected from light between measurements. For CLSM, a Leica DMi8 microscope (Leica, Wetzlar, Germany) stand equipped with a SP8 scanning head, a 405 nm laser, and a tunable white light laser (470 to 670 nm), equipped with 2 HyD and 2 PMT detectors, was used. A 40× oil immersion objective was employed, and excitation and emission wavelengths were optimized for the different staining dyes.

### 4.4. Data Evaluation

**Figure 5:**
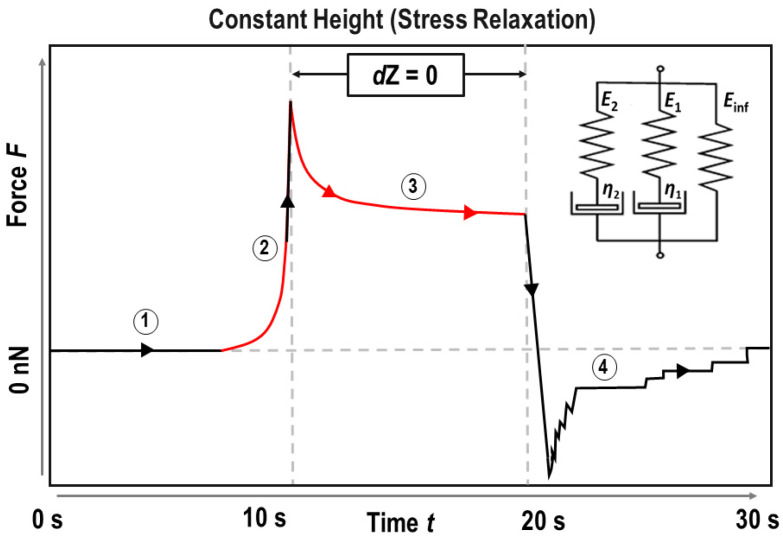
Illustrative stress–relaxation AFM curve. Section (1) marks the baseline at approach, (2) the indentation segment with the Hertz-fitted range in red, (3) the constant height (stress relaxation) segment onto which a double exponential decay function is used to fit the data, and (4) the retract segment with adhesion spikes. The inset shows a graphical representation of the five-element Maxwell model (Zener’s model) with a spring in parallel with two spring-dashpot elements which was used to derive the viscoelastic properties (3).

All analyses of force spectroscopy curves were performed using the R afmToolkit, developed by Benitez et al. in our laboratory [73]. Force–distance curves were imported into the toolkit, contact and detachment points were determined, baseline subtraction was performed, and the zero-force point was determined. Young’s moduli were calculated by fitting Equation (1), the Sneddon–Hertz model,

(1) $$F=\frac{4}{3}\sqrt{R_{C}}\frac{E_{app}}{1-\nu^{2}}\delta^{\frac{3}{2}},$$

onto the first 500 nm of the indentation segment. Restricting the fit to the first 500 nm of indentation allowed for the assumption of linear elasticity to be met [37]. Here, *R_c_* denotes the radius of the spherical indenter (here: particle Ø = 10 µm), while *E_app_* is the elastic (Young’s) modulus. The Poisson ratio *ν* was assumed to be 0.5 for an incompressible fluid and *δ* denotes the deformation of the sample under load. Considering the low applied forces, the shallow indentation depths and the short fitting range onto the indentation curve, the contact profile for a paraboloid indenter was chosen over a spherical contact model (for details see Figure S5 of the Supplementary Information). Viscoelastic properties were calculated via a five-element Maxwell model (Zener’s model), a graphical representation of which is found in Figure 5, by fitting a double exponential decay function (Equation (2)) onto the stress–relaxation segment:

(2) $$F=A_{0}+A_{1}e^{-\frac{t}{\tau_{1}}}+A_{2}e^{-\frac{t}{\tau_{2}}},$$

From these, the (elastic) moduli *E*_inf_, *E*_1_ and *E*_2_ are derived with consideration of the sample deformation by modeling the stress relaxation as:

(3) Ft=C1−νEinf+E1e−tτ1+E2e−tτ2,

with *C* being a geometry-dependent constant defined as:

(4) $$C=\frac{4}{3}\sqrt{R\delta_{0}^{3}}.$$

The viscosities *η*_1_ and *η*_2_ were calculated from the relaxation times and the associated moduli. A detailed derivation of the mechanical model and the calculation for each parameter is found in the Methods section of our previous publication [72].

Confocal images were processed using ImageJ/FIJI software (version 2.9.0, National Institute of Health, Madison, WI, USA) [74] to adjust brightness and contrast, add scale bars and assign lookup tables.

For statistical analysis, Origin Pro 2018 (OriginLab, Northampton, MA, USA) software was used. Outliers were detected via Grubbs testing and removed. Significances were determined via ANOVA. Levels of significances are indicated as ‘***’ for *p* < 0.001, ‘**’ for *p* < 0.01, ‘*’ for *p* < 0.5. All box plots show boxes ranging from the 25th to 75th percentile and whiskers ranging from the 10th to 90th percentile.

## Figures and Tables

**Table 1:** Mean values of the elastic and viscoelastic properties derived from AFM force spectroscopy measurements on different breast cancer cell lines.

	MCF10A	MCF-7	MDA-MB-231	TamR
*E*_elastic_ [Pa]	279	249	224	148
*δ* [µm]	0.8	1.1	0.9	1.4
*E*_inf_ [Pa]	104	79	105	42
*E*_1_ [Pa]	144	81	83	40
*E*_2_ [Pa]	76	58	74	23
*E*_inst_ [Pa]	323	218	262	113
*τ*_1_ [s]	0.13	0.16	0.18	0.19
*τ*_2_ [s]	2.94	3.46	4.21	3.78
*η*_1_ [Pa s]	17	12	13	7
*η*_2_ [Pa s]	224	204	340	114

## Data Availability

Data are available from the corresponding author upon request.

## Supplementary Materials

The supporting information can be downloaded at: https://www.mdpi.com/article/10.3390/ijms241512208/s1.
