# Supplementary material for: Characterization of Breast Cancer Aggressiveness by Cell Mechanics
Source: Int J Mol Sci. 2023 Jul 30;24(15):12208. doi: 10.3390/ijms241512208 (PMC10418463; doi:10.3390/ijms241512208)
Supplement: Supplementary file 1 [file ijms-24-12208-s001.zip › ijms-2494401-supplementary.pdf]

Table S1. Detailed breakdown of the elastic and viscoelastic parameters listed in **table 1** of the main text. Mean and median values including standard error of the mean, standard deviation and sample size for each cell line and each parameter.

**Young's Modulus**	**N**	**Mean [Pa]**	**SEM [Pa]**	**STD [Pa]**	**Median [Pa]**
**MCF10A**	418	279	9.9	196	236
**MCF-7**	474	249	5.4	118	228
**MDA-MB-231**	289	224	8.3	141	190
**TamR**	168	148	7.0	90	126
**Indentations** δ	**N**	**Mean [µm]**	**SEM [µm]**	**STD [µm]**	**Median [µm]**
**MCF10A**	418	0.8	0.01	0.3	0.8
**MCF-7**	474	1.1	0.02	0.4	1.0
**MDA-MB-231**	290	0.9	0.02	0.3	0.9
**TamR**	168	1.4	0.03	0.4	1.4
E_{inf}	**N**	**Mean [Pa]**	**SEM [Pa]**	**STD [Pa]**	**Median [Pa]**
**MCF10A**	423	104	5	103	67
**MCF-7**	289	79	3	443	72
**MDA-MB-231**	202	105	5	78	87
**TamR**	70	42	2	18	37
E_1	**N**	**Mean [Pa]**	**SEM [Pa]**	**STD [Pa]**	**Median [Pa]**
**MCF10A**	423	144	5	100	113
**MCF-7**	289	81	3	55	62
**MDA-MB-231**	202	83	4	52	71
**TamR**	70	40	2	13	36
E_2	**N**	**Mean [Pa]**	**SEM [Pa]**	**STD [Pa]**	**Median [Pa]**
**MCF10A**	423	76	2	46	63
**MCF-7**	289	58	2	38	48
**MDA-MB-231**	202	74	3	46	65
**TamR**	70	23	1	11	28
E_{inst}	**N**	**Mean [Pa]**	**SEM [Pa]**	**STD [Pa]**	**Median [Pa]**
**MCF10A**	423	323	11	230	225
**MCF-7**	289	218	7	125	185
**MDA-MB-231**	202	262	11	158	229
**TamR**	70	113	5	39	104
η_1	**N**	**Mean [Pa s]**	**SEM [Pa s]**	**STD [Pa s]**	**Median [Pa s]**
MCF10A	423	17	0.5	9.2	14
MCF-7	289	12	0.5	7.9	10
MDA-MB-231	202	13	0.6	8.2	12
TamR	70	7	0.4	3.2	7
η_2	**N**	**Mean [Pa s]**	**SEM [Pa s]**	**STD [Pa s]**	**Median [Pa s]**
MCF10A	423	224	8	166	173

MCF-7	289	204	10	172	144
MDA-MB-231	202	340	20	287	252
TamR	70	114	8	66	101
τ_1	**N**	**Mean [s]**	**SEM [s]**	**STD [s]**	**Median [s]**
MCF10A	423	0.13	0.01	0.05	0.12
MCF-7	289	0.16	0.01	0.06	0.16
MDA-MB-231	202	0.18	0.01	0.07	0.15
TamR	70	0.19	0.01	0.06	0.18
τ_2	**N**	**Mean [s]**	**SEM [s]**	**STD [s]**	**Median [s]**
MCF10A	423	2.94	0.1	1.5	2.7
MCF-7	289	3.46	0.1	1.6	3.1
MDA-MB-231	202	4.21	0.2	1.7	4.0
TamR	70	3.78	0.2	2.2	3.5

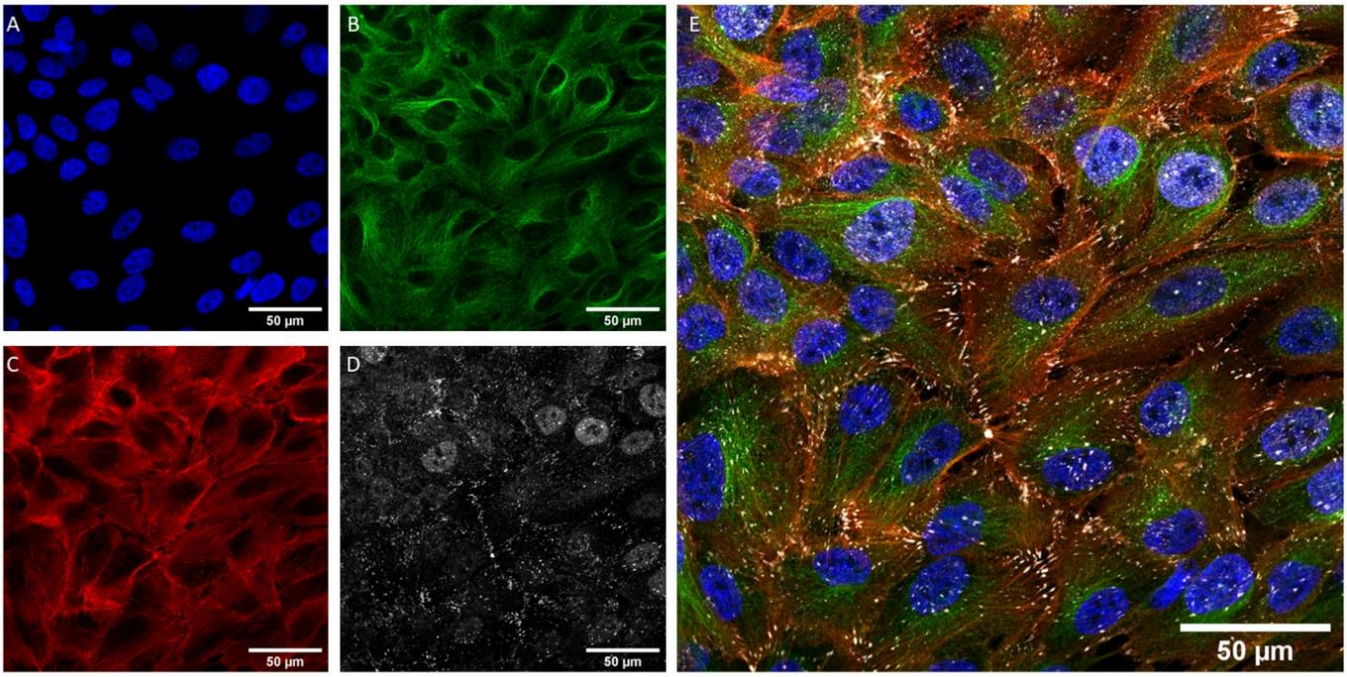

Figure S1. Representative immunofluorescence CLSM images of MCF10A cells: (A) shows nuclei in blue, (B) microtubules in green, (C) the actin cytoskeleton in red, (D) vinculin in grey and (E) a composite overlay. The scale bar corresponds to 50 µm.

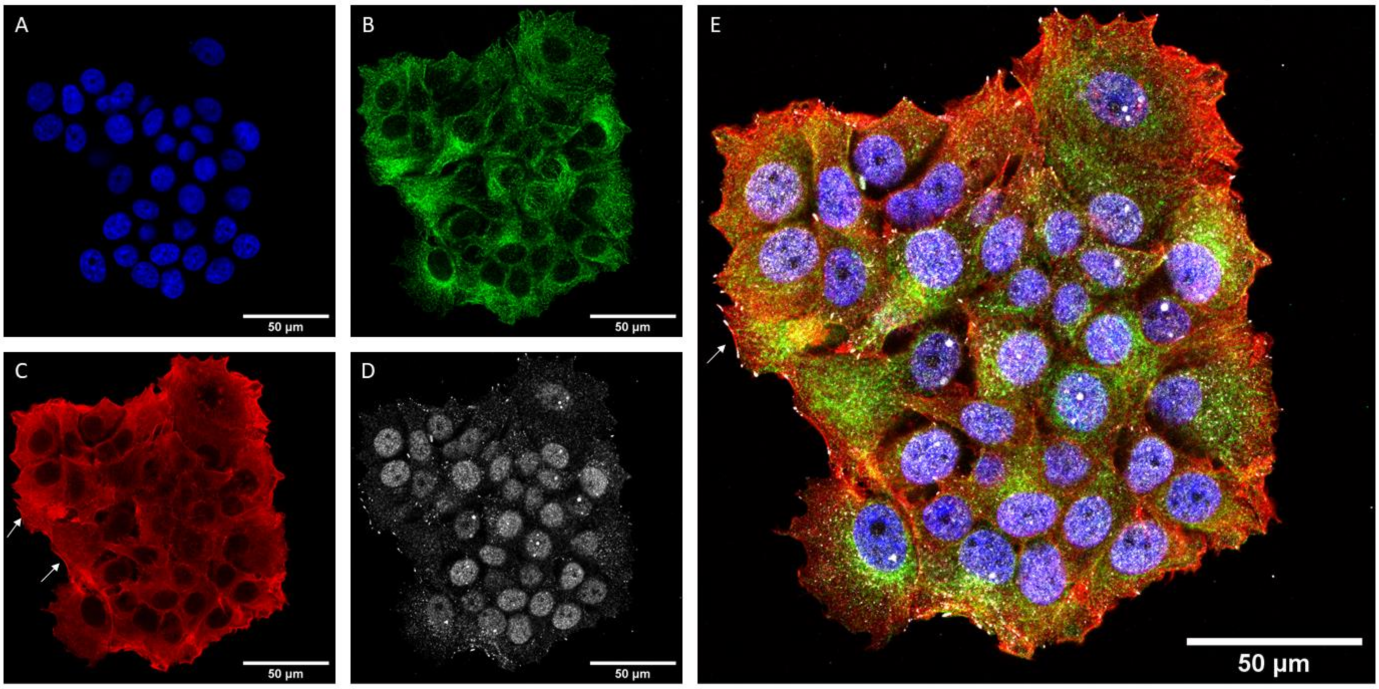

Figure S2. Representative immunofluorescence CLSM images of MCF-7 cells: (A) shows nuclei in blue, (B) microtubules in green, (C) the actin cytoskeleton in red, (D) vinculin in grey and (E) a composite overlay. The arrows in (C) indicate stress fibers ending in focal adhesions. Such structures seem absent from MCF-7TamR. The scale bar corresponds to 50 μm.

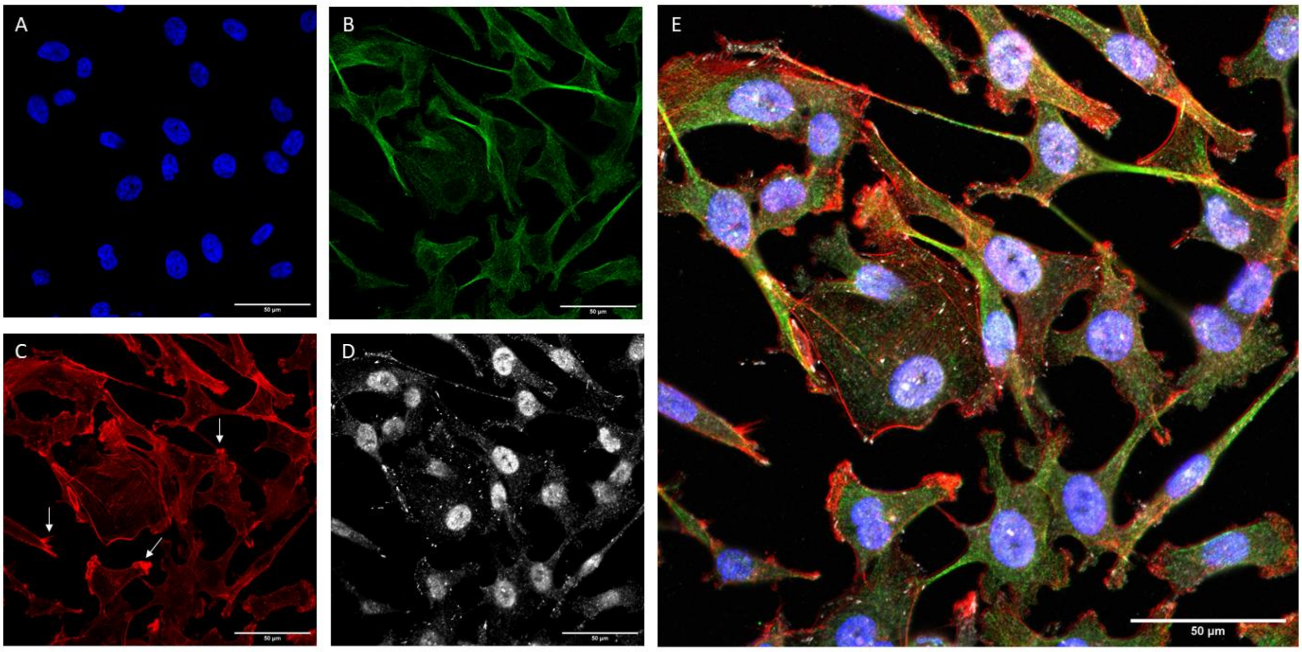

Figure S3. Representative immunofluorescence CLSM images of MDA-MB-231 cells: (A) shows nuclei in blue, (B) microtubules in green, (C) the actin cytoskeleton in red, (D) vinculin in grey and (E) a composite overlay. In (C) and (E) migratory actin protrusions are visible at the borders of the cells, which do not associate with focal adhesions (D). MDA-MB-231 show the most heterogeneity in cell morphology among the studied cell lines and are regarded the most invasive. The arrows in (c) indicate distinct types of migratory actin protrusions. The scale bar corresponds to 50 μm.

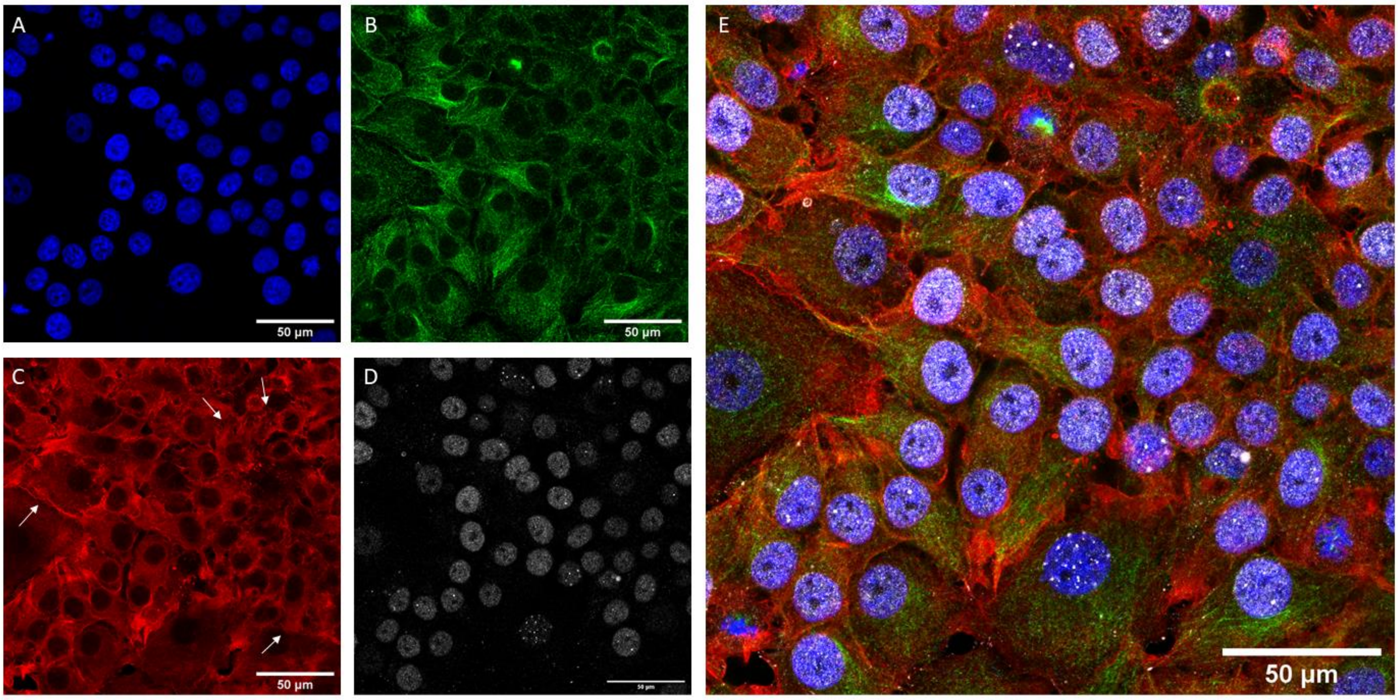

Figure S4. Representative immunofluorescence CLSM images of MCF-7TamR cells: (A) shows nuclei in blue, (B) microtubules in green, (C) the actin cytoskeleton in red, (D) vinculin in grey and (E) a composite overlay. The frizzier actin structures compared to MCF-7 can be seen in (C) indicated with arrows and (E), as well as the decreased number of focal adhesions (D). This hints at a more motile phenotype. No remarkable difference is visible in tubulin arrangement. The scale bar corresponds to 50 µm.

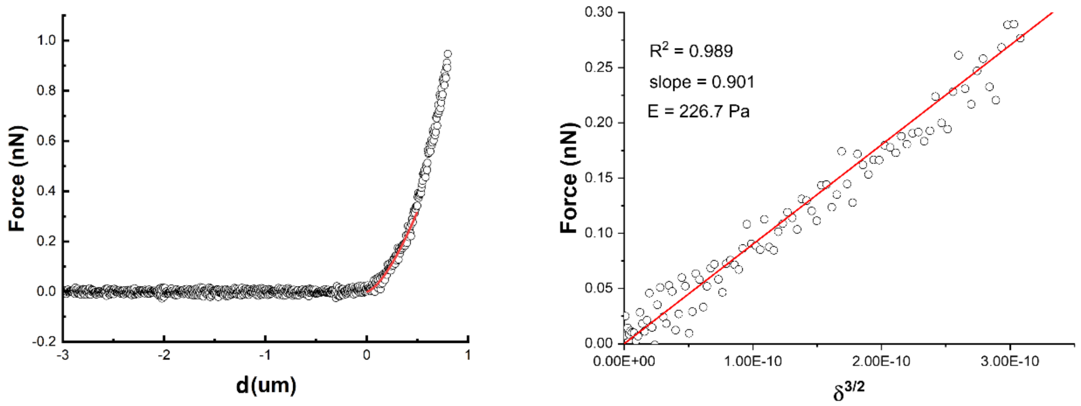

Figure S5. Left: representative force-distance curve. The fitting range in the curve is marked in red. Right: representative fitting using equation 1 (force vs. $\delta^{3/2}$). Note that (δ) denotes indentation. In this case, the obtained elastic modulus is about 226.7 Pa (with a coefficient of determination R^2 closed to 0.99).
